# Association of high pre-pregnancy body mass index with adverse maternal and perinatal outcomes

**DOI:** 10.12669/pjms.40.3.7317

**Published:** 2024

**Authors:** Qudsia Qazi, Nazia Liaqat, Fauzia Afridi, Saima Khattak

**Affiliations:** 1 Dr. Qudsia Qazi, FCPS. Associate Professor, Department of Gynae, Medical Teaching Institute, Lady Reading Hospital, Peshawar, Pakistan; 2 Dr. Nazia Liaqat, FCPS. Assistant Professor, Department of Gynae, Medical Teaching Institute, Lady Reading Hospital, Peshawar, Pakistan; 3 Dr. Fouzia Afridi, FCPS. Assistant Professor, Department of Gynae, Khyber Teaching Hospital, Peshawar, Pakistan; 4 Dr Saima Khattak, FCPS. Assistant Professor, Department of Gynae, Medical Teaching Institute, Lady Reading Hospital, Peshawar, Pakistan

**Keywords:** Pre pregnancy BMI, Postpartum hemorrhage, Fetal macrosomia

## Abstract

**Background & Objectives:**

Obesity is an epidemic of the 21^st^ century with its rates doubling in both developed and developing countries. It raises concerns for both maternal and fetal well-being and needs altered care throughout pregnancy and in postnatal period. Raised BMI prior to conception is associated with adverse feto-maternal outcomes. Limited data is available about its association with adverse maternofetal outcome in this region of the world. Our objective was to find out association of high pre-pregnancy BMI with adverse maternal and perinatal outcomes.

**Methods:**

This cohort study of 390 patients was conducted in Gynae department of Lady Reading Hospital Peshawar. Study duration was from June 2021 to March 2022. Patients enrolled in third trimester of gestation (≥ 37 weeks) were divided into two groups based on BMI i.e., Group-A with BMI <25 and Group-B with BMI ≥ 25. Both groups were followed until their delivery and discharge.

**Results:**

The mean age of 390 women was 28.2 ± 4.8 years. There was statistically significant association between raised pre pregnancy BMI and maternal risks like postpartum hemorrhage (p-0.0001), genital tract (p-0.0002) and perineal trauma (p-0.04). Neonatal risks significantly associated with high pre-pregnancy BMI were macrosomia (p-0.0001), and one minute APGAR score of < 8/10(p- 0.01). Both groups had no statistically significant difference for different modes of delivery i.e normal vaginal/ instrumental delivery and cesarean section (P-value 0.9).

**Conclusion:**

Maternal pre-conception BMI of ≥ 25 leads to poor maternal and perinatal outcomes.

## INTRODUCTION

High pre-conception body mass index is viewed as key health concern and has been declared as pandemic issue by WHO. In a Turkish study the prevalence of obesity and overweight in pregnancy was 48%.^1^ It is mostly observed amongst women of reproductive age i.e., 14% of women compared to 10% of men. Its overall recent estimated rate is between 18% - 25.3% in many countries.^1^ Worldwide more than 21% of women will be obese in the year 2025, with yearly expected rise.^2^,^3^ Burden of obesity and overweight in married women was 11-15% according to an Indian Health Survey 2005–2006 (NFHS) 3, which rose to 20.6% as per NFHS 4.^4^

Steroidal peptide hormones and adipokines produced by adipose tissue (like adiponectin, adipsin, cytokines and acylation-stimulating protein) cause dysregulation of certain tissue functions like uterine contractility with increased risk of prolonged pregnancy, dysfunctional labor, operative delivery and postpartum haemorrhages.^5^,^6^ Leptin and adiponectin secreted by adipose tissue play a key role in metabolic and inflammatory responses due to interaction of insulin with other tissues. High angiotensin and TNF-α level in obesity cause endothelial dysfunction due to raised blood pressure and thrombi with the resultant worst obstetric outcomes like preeclampsia, fetal distress, preterm labor and/or caesarean section.

 Increased prevalence of obesity is due to factors like social demographics, different health behavior practices, attitudes about dietary knowledge and physical activity. A well-accepted fact is that weight before pregnancy is more important than antepartum weight gain regarding risk of complications. Obesity associated adverse events are gestational diabetes and hypertension, thromboembolic disorders, perineal trauma, postpartum hemorrhage, cesarean section and maternal mortality. Various neonatal risks reported by many studies are abortions, congenital defects, fetal macrosomia and dys-maturity, shoulder dystocia, birth trauma, decreased Apgar scores, neonatal respiratory distress and meconium aspiration syndromes, high perinatal mortality rate.

Other adverse outcomes are reduced rate of successful vaginal delivery, cesarean section, intra-operative complications like increased infectious morbidity, labor induction, instrumental delivery, close post term fetal surveillance; neonatal intensive unit admission and ventilator use.

A dose-dependent relationship has been found between higher pre pregnancy BMI and fetal macrosomia (an estimated fetal weight of ≥ 4500 g), with a two-to-three-fold increased rate seen in obese women.^7^,^8^ Postpartum risks are frequently seen in obese women like infected wound, depressive disorders and poor breastfeeding.^9^,^10^

Body mass index is a best ever available tool used to easily calculate and measure obesity. The World Health Organization (WHO) BMI categorizing criteria is specified as normal weight with (18.5–24.9 kg /m^2^) while obese as (≥30.0 kg/ m^2^). A body mass index of 25.0-29.9 is defined as overweight according to WHO. Further obesity subclasses are class I (30.0–34.9 kg /m^2^), class II (35.0–39.9 kg /m^2^) and class III (≥ 40 kg /m^2^). According to Asian specific criterion normal weight is ═ 18.5–23 kg/ m^2^, overweight ═ 23–27.5 kg /m^2^ and obese ═ >27.5 kg/ m^2^, as the Asians are susceptible to develop metabolic diseases at relatively lower BMI.^11^,^12^

The objective of the current study was to determine association of high preconception BMI with adverse maternal and perinatal outcomes. The rationale of the study was paucity of prospective studies on the subject from our part of the world. Findings of this study will hopefully provide valuable addition to existing pool of evidence and will open window for further larger studies.

## METHODS

This cohort study was conducted in gynecology department of Lady Reading Hospital Peshawar from June 2021 to March 2022. A sample size of 390 women was calculated via online Open-Epi sample size calculator. Power of test was taken as 80%, confidence level of 95%, proportion of macrosomia in unexposed as 2% versus 8%^12^ in exposed. Weight (pre-pregnancy weight in kg) was divided by height in (m^2^) to calculate BMI.

### Ethical Approval

The study was approved by hospital ethical committee (IREB Ref No:130 /MTI/LRH dated 28/5/21).

### Inclusion Criteria:

- Data available about woman’s pre pregnancy BMI of 18 kg/m^2^ - 35 kg/m^2^ (taken from documented first trimester antenatal booking record at ≤ 13 weeks of gestation),
- Women at term (37-40 weeks),
- Single alive, cephalic fetus,
- low risk.

### Exclusion Criteria:

- Fetal anomalies,
- Maternal medical comorbidities e.g., pre-pregnancy diabetes or chronic hypertension.

Enrolled women were categorized into two groups based on their pre pregnancy BMI. Group-A had BMI from 18-24.9 kg/m^2^ and Group-B had patients with BMI from 25- 35 kg/m^2^. Recruitment was done by using non probability consecutive sampling technique and pre-defined inclusion and exclusion criteria. Women were then followed up in antenatal clinic on weekly basis until their delivery in our department and postpartum outcomes until discharge were studied. Informed consent was obtained from participants of the study.

Maternal characteristics included age, gravidity, parity, mode of delivery, maternal postpartum complications i.e., Hemorrhage ≥ 500 mL, perineal, cervical or vaginal tears. Neonatal outcomes observed were fetal distress (assessed by CTG), Apgar score at One minute < 8/10, macrosomia (defined as birth weight ≥ 4000 Kg**)** and necessity for transfer to NICU. Detailed history and clinical examination were done to evaluate all women. Maternal and fetal outcomes were recorded in predefined proforma. Mean and standard deviation were computed for quantitative variables like maternal age, gestational age, maternal weight, height and BMI. Percentages were calculated for qualitative variables like parity, mode of delivery, post-partum maternal and perinatal outcomes. P-value of < 0.05(two-tailed) was selected to be significant. SPSS version 23 (IBM-SPSSV-23) was used to analyze data.

## RESULTS

Out of 390 participants, 135 were with BMI ≥ 25 and 255 with < 25. The mean age of women was 28.2 ± 4.8 years. Extreme age groups of 18-20 years and 39-42 years had statistically significant association with BMI (P-value 0.04 and p- 0.001 respectively). Comparison of various modes of delivery between two groups is shown in Table-II. There was no association between cesarean section and pre-pregnancy BMI when controlled for other confounders. (p- 0.9, adjusted Odd Ratio :1.93, 95% CI:0.12-6.87).

**Table-I:** Demographic Features of the study participant..

AGE	≥25 BMI	<25BMI	Total	P-Value
18-21 yrs.	8(5.9%)	32(12.5%)	40(10.2%)	0.04
22-25 yrs.	30(22.2%	71(27.8%)	101(25.8%)	0.22
26-29 yrs.	30(22.2%)	51(20.0%)	81(20.7%)	0.26
30-34 yrs.	27(20%)	62(24.3%)	89(22.8%)	0.33
35-38 yrs.	28(20.7%)	36(14.1%)	64(16.4%)	0.09
39-42 yrs.	12(8.9%)	3(1.2%)	15(3.8%)	0.0001

Total	135(34.6%)	255(65.4%)	390(100%)	

**Table-II:** Association of BMI and mode of delivery.

	<25 BMI	≥25 BMI	TOTAL	p-values	aOR	95%CI
Normal vaginal delivery	239 (61.3%)	77 (19.7%)	316 (81.0%)			
Instrumental delivery	8 (2.1%)	19 (4.9%)	27 (6.9%)	0.0005	1.3	0.2-13
Cesarean section	8 (2.1%)	39 (10.0%)	47(12.1%)	0.0001	1.9	0.12-6.87

TOTAL	255 (65.4%)	135 (34.6%)	390 (100%)			

Statistically significant association (P-value < 0.001) between raised pre-pregnancy BMI and postpartum hemorrhage (aOR;9.8, 95% CI:2.1-44.5, p-0.0001) and genital tract injuries (aOR;3.7, 95% CI:1.48-29.5, p-0.0002) is shown in Table-III. Association of raised pre-pregnancy BMI (i.e., ≥ 25) with macrosomia, low APGAR score and increased NICU admission is shown in Table-IV.

**Table-III:** BMI AND Maternal Outcomes.

Maternal Outcomes	<25 BMI	≥25BMI	Total	P-Value	a OR	95%CI
No complication	255 (65.4%)	105 (26.9%)	360 (92.3%)	<0.001		
Postpartum Hemorrhage	09(2.3%)	18 (4.6%)	27 (20.3%)	0.0001	9.8	2.1-44.5
Genital tract injuries	01(0.25%)	11(2.8%)	12(3.1%)	0.0002	3.7	1.48-29.5

Total	255 (65.4%)	135 (34.6%)	390 (100%)			

**Table-IV:** BMI AND Fetal Outcome.

Fetal outcome	BMI <25	BMI ≥ 25	Total	P-value	aOR	95% CI
No complications	247 (63.3%)	109 (27.9%)	356 (91.3%)	<0.001		
Macrosomia	1 (0.09%)	14 (3.6%)	14 (3.6%)	0.0001	8.40	0.66-107
Fetal Distress	8 (2.1%)	12 (3.1%)	20 (5.1%)	0.06		
APGAR score						
>8 / 10	8 (2.1%)	4 (1.0%)	12 (3.1%)	0.09		
= 8/ 10	239 (61.3%)	104 (26.7%)	343 (87.9%)			
<8/ 10	8 (3.1%)	27 (20.0%)	35(9.0%)	0.01	7.5	1.3-40.8
NICU admission	5(1.96%)	17(12.59%)	22(14.55%)	<0.0001	57.9	12.7-82.3

Total	255 (65.4%)	135 (34.6%)	390 (100%)			

## DISCUSSION

This prospective cohort study detected significant association of high maternal pre-pregnancy BMI with adverse maternal and perinatal outcomes like PPH, genital tract injuries, low APGAR score, NICU admissions and macrosomia.

Ozdilek R et al. emphasized upon importance of pre-pregnancy counselling for weight loss as it directly affects weight gain during pregnancy and feto-maternal outcomes.^13^ Most epidemiological studies regarding this association are mainly from Western countries with limited literature from Asian population. A systematic review by Goldstein reported higher pre pregnancy BMI in women from Western countries than eastern Asians.^14^

In our study, women with BMI of ≥25 were mostly within age group of 22-38 years with mean age of about 28.2 ± 4.8 years. Our result was comparable with those of junita with a mean age of cases being 32 years, showing that overweight or obesity at a rather early age predisposes women to high-risk pregnancies.^15^

Our observation of association of high pre-pregnancy body mass index with adverse pregnancy outcomes are consistent with those of Salmon et al and Munim showing a higher ratio of caesarean section for various obesity classes (37.2% for obesity class I;43.4% for obesity class II; and 52.2% for class III or more).^16^,^17^ Liu B et al. at Taiwan (adjusted risks [aOR-2.66, CI 95%, 2.11-3.36] and Junita (caesarean section in 86.5% of obese women compared to 10.8% delivered vaginally) observed higher cesarean section rate with excessive gestational weight gain than controls.^15^,^18^ These findings can be due to inherent differences (due to race and ethnicity) in local rates and indications of caesarean section. In addition, they included all classes of obesity (>30 kg/m^2^) while we restricted our criteria to highest BMI of 35 kg/m^2^.

Hung et al. and Kutchi et al. reported comparable results for poor progress of labor (adjusted OR 1.47, 95% CI 1.03–2.11), atonicity and peri and post-cesarean section hemorrhage (22.4%, OR:0.19; 0.03-1.36) in obese compared to normal weight women. Results for fetal macrosomia are comparable to ours i.e., 19.5% vs 3.6% cases of large for gestational age fetuses in obese versus 8.2% vs 0% of normal weight women.^12^,^19^

Hung et al. and others observed neonatal one minute APGAR score of < 7/10 in 2.2% of obese women compared to 20% in this study.^19^ High pre conception BMI was reported to have significant association with postpartum hemorrhage (OR: 1.39; 95% CI: 1.32-1.46) and genital tract infection (OR: 1.30; 95% CI: 1.07-1.56) in a study conducted by Sabire.^20^

Another study by Sonia found no statistically significant association between obesity (at first prenatal visit) and duration of labor, postpartum hemorrhage, Apgar scores at one and five minutes after birth, early neonatal mortality, and maternal and fetal injury.^21^

Our results of significant association of perineal tears with obesity are comparable to those of Kundu showing significant positive correlation between pre-pregnancy BMI and BMI at delivery and the incidence of genital tract trauma. (k = 0.010, p = 0.02).

Kundus also found a positive correlation (r = 0.009, p = 0.03) between BMI at the time of delivery and fetal macrosomia. This analysis also demonstrated significant correlation between the rate of a low pH < 7.14 at birth and higher BMI either pre-conception (r = 0.017, p = 0.021) or at the time of childbirth (r = -0.014, p = 0.071).^22^

Macrosomia is due to metabolically rich environment with raised blood sugar and lipids delivery to fetus. This result in hyperinsulinemia, transplacental glucose gradient, altered fetal lipid metabolism and disproportionate fetal adipose tissue deposition.^23^ It signifies the importance of individualized pre-gestational nutritional plan to ensure optimal fetal growth. Sebire et al (OR:1.57 (1.50--1.64) and Chi-Nien Chen (5.23%) also observed increased risk of macrosomia with obesity.^20^,^24^

Papachatzi and John observed association of maternal obesity (cases vs control) with increased need for IOL (20% vs 4%), caesarean section (38%vs 12%) and NICU admissions for stabilization of the newborns (22% vs 10%, p = 0.012). Reason for majority of these admissions (12% of 22%) was fetal distress.^25^,^26^ Our results of NICU admission (12.5 % vs 1.9%) were consistent with reports of Junita.^15^ ACOG clinical showed NICU admissions of about 35.7% for obese women, the reason for admission being jaundice, hypoglycemia, perinatal distress, birth defects, and congenital anomalies.^27^ Multivariate analysis has shown that correlation between factors like social status, genetic and sociocultural factors should also be considered.^28^

### Limitations

Our study design of prospective cohort study is strength of our study while its limitations are that it is a single center study with less cases and we had to rely on patients booking BMI as substitute for pre pregnancy BMI as every woman did not know how much they weigh right before their pregnancy. The maternal BMI is certainly not the only factor to influence adverse perinatal outcome.

## CONCLUSION

High maternal pre pregnancy BMI is significantly associated with adverse maternal and perinatal outcomes.

### Recommendation

Pre conception or early conception period is the most appropriate time to investigate obesity and find solution for it. Our study findings highlight the importance of educating women of reproductive age about optimal pre pregnancy BMI. Prenatal counselling for optimal gestational weight gain (GWG), appropriate multidisciplinary management of obese mothers and their fetus can optimize pregnancy outcomes.

### Author Contribution:

**QQ:** Conceived idea, did data collection and prepared the manuscript, responsible and accountable for the accuracy and integrity of the work.

**NL:** Designed, did statistical analysis, edited and finalized manuscript, responsible and accountable for the accuracy and integrity of the work.

**FA:** Did literature search and reviewed manuscript.

**SK:** Data collection, literature search, editing of manuscript.
